# Depot-Specific White Adipose Tissue Remodeling Supports Non-Thermogenic Metabolic Homeostasis During Shallow Hibernation in Raccoon Dogs

**DOI:** 10.3390/ijms27125611

**Published:** 2026-06-22

**Authors:** Ruojun Zong, Zhiqiang Han, Runzhou Liu, Manman Yang, Xin Liu, Xiuli Zhang, Jiahao Hu, Rui Du, Chao Xu

**Affiliations:** 1 College of Animal Science and Technology, Jilin Agriculture University, Changchun 130118, China; zongruojun20@mails.ucas.ac.cn (R.Z.); hanzhiqiang5113@163.com (Z.H.); ymm200207@163.com (M.Y.); liu6428y@163.com (X.L.); xiuli23@jlu.edu.cn (X.Z.); 2 State Key Laboratory of Organ Regeneration and Reconstruction, Institute of Zoology, Chinese Academy of Sciences, Beijing 100101, China; liurunzhou23@ioz.ac.cn (R.L.); hujiahao0720@gmail.com (J.H.); 3 College of Agriculture, Yanbian University, Yanji 133002, China

**Keywords:** raccoon dogs (*Nyctereutes procyonoides*), hibernation, adipose tissue

## Abstract

White adipose tissue (WAT) is essential for maintaining energy homeostasis during hibernation by supplying lipolysis-derived fatty acids as a major fuel source. In raccoon dogs (*Nyctereutes procyonoides*), the activity of brown adipose tissue is diminished, providing a unique model to investigate how WAT supports metabolic homeostasis in a largely non-thermogenic state. Here, we integrated physiological, histological, transcriptomic, and molecular analyses of back-fat and tail-fat depots during autumn fattening and winter sleep. Despite reduced food intake, body weight loss, and mild hypothermia, raccoon dogs maintained systemic glucose and lipid homeostasis. Both WAT depots exhibited adipocyte atrophy and the coordinated suppression of core metabolic and biosynthetic pathways, indicating a shared program of metabolic depression. However, the two depots adopted distinct remodeling strategies. Back-fat showed collagen densification and vascular-associated remodeling, suggesting a structural adaptation that may preserve tissue integrity during winter sleep. In contrast, tail-fat displayed enhanced innate immune signaling and M2 macrophage enrichment, indicating immune niche remodeling that may support tissue protection during prolonged lipid mobilization. Together, these findings reveal that raccoon dogs maintain metabolic homeostasis during shallow hibernation through a non-thermogenic, WAT-centered strategy characterized by shared metabolic depression and depot-specific structural and immunometabolic remodeling.

## 1. Introduction

White adipose tissue (WAT) is a highly dynamic metabolic and endocrine organ that plays essential roles in energy storage, immune regulation, extracellular matrix remodeling, and systemic metabolic homeostasis [1,2]. Under prolonged metabolic stress, WAT must coordinate energy release with tissue integrity to avoid pathological inflammation, fibrosis, or metabolic dysfunction [3,4]. Natural hibernation provides a unique physiological context in which mammals endure extended periods of fasting, reduced energy expenditure, and seasonal cold exposure while preserving metabolic homeostasis and tissue function [5,6]. Therefore, hibernating mammals represent valuable models for understanding how WAT undergoes adaptive, non-pathological remodeling under extreme metabolic conditions.

The raccoon dog (*Nyctereutes procyonoides*) is a canid species that undergoes autumnal fattening followed by winter sleep [7,8]. Unlike classical deep hibernators such as ground squirrels and marmots [9], the raccoon dog undergoes a shallow form of hibernation, characterized by a modest reduction in core body temperature of only 0.5–1.5 °C and relatively stable metabolic rates [7,10,11,12,13,14]. This hibernation strategy more closely resembles that of large-bodied hibernators such as bears [15], yet the raccoon dog’s smaller body size and ease of handling make it an attractive model for investigating metabolic flexibility and tissue adaptation [16,17].

Adipose tissue plays a central role in energy metabolism during hibernation [6,18,19], with hibernating mammals depending on fatty acids derived from lipolysis as their primary fuel source [5]. In raccoon dogs, the winter season is preceded by a period of autumnal hyperphagia, during which raccoon dogs feed extensively, increasing body weight by 30–40% through WAT accumulation [20]. These abundant WAT stores enable raccoon dogs to sustain fasting periods of up to 11 weeks [21].

Previous research of hibernation metabolism mainly focused on the thermogenic function of brown adipose tissue (BAT) [22,23]. However, in raccoon dogs, PET/CT imaging revealed no detectable BAT activity during winter, and uncoupling protein 1 (*UCP1*) expression is ineffectively induced in cultured adipocytes [24]. These findings suggest that BAT thermogenic capacity is substantially diminished in this species, shifting the burden of energy maintenance and cold defense toward WAT. Interestingly, a multi-tissue transcriptomic study in grizzly bears demonstrated that the number of differentially expressed genes (DEGs) in WAT far exceeds that in liver and skeletal muscle, indicating that WAT experiences the most extensive transcriptional reprogramming of any tissue [15]. The identified changes include the broad suppression of metabolic pathways, reprogramming of fatty acid metabolism, and downregulation of insulin signaling, underscoring the central role of WAT in maintaining metabolic homeostasis during winter [15]. However, the molecular landscape of WAT in the raccoon dog during hibernation remains unclear.

Here, we performed RNA-sequencing to characterize the transcriptomic profiles of back-fat and tail-fat in raccoon dogs during hibernation compared with the autumn fattening phase. Our analysis reveals distinct adaptive strategies employed by these two fat depots. This study provides new insights into the molecular mechanisms underlying WAT adaptation during shallow hibernation and offers a foundation for understanding the depot-specific functional divergence of adipose tissue under extreme physiological conditions.

## 2. Results

### 2.1. Raccoon Dogs Maintain Systemic Homeostasis During Shallow Hibernation

**Figure 1:**
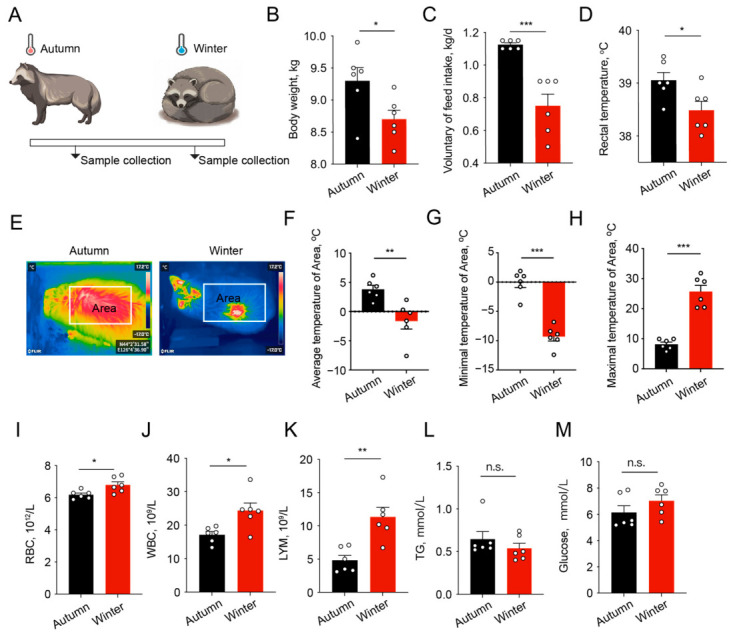
Characterization of physiological and metabolic shifts in raccoon dogs during winter sleep. (**A**), Experimental design and sample collection timeline for the autumn fat-accumulation phase and winter sleep phase. (**B**–**D**), Seasonal changes in primary physiological parameters, including body weight. (**B**), voluntary feed intake (**C**), and rectal temperature (**D**), *n* = 6. (**E**), Representative infrared thermography images of raccoon dogs during autumn and winter, highlighting changes in heat distribution and postural conservation. (**F**–**H**), Quantitative analysis of infrared thermography data: average temperature (**F**), minimal temperature (**G**) and maximal temperature (**H**) of the observed body area, *n* = 6. (**I**–**K**), Hematological profiling showing red blood cell (RBC) count (**I**), total white blood cell (WBC) count (**J**) and absolute lymphocyte count (LYM) (**K**), *n* = 6. (**L**,**M**), Serum biochemical levels of triglycerides (TG) (**L**) and glucose (**M**), *n* = 6. Data are presented as mean ± SEM, * *p* < 0.05, ** *p* < 0.01, *** *p* < 0.001, n.s. means not significant.

To evaluate the physiological status of raccoon dogs during seasonal transition, we monitored body weight, feed intake, and temperature fluctuations from the autumn fat-accumulation phase (autumn) to the winter sleep phase (winter) (Figure 1A). Because raccoon dogs accumulate fat most actively during October to November [25], samples collected in October were used to represent the autumn fat-accumulation phase. The January sampling point was selected to represent the stable winter sleep phase after seasonal reductions in food intake and body weight had occurred. Compared with the autumn fat-accumulation phase, raccoon dogs in the winter sleep phase exhibited significant reductions in both body weight and voluntary feed intake (Figure 1B,C). Although raccoon dogs were not in deep torpor, their rectal temperatures showed a slight but significant decrease from approximately 39 °C to 38.5 °C (Figure 1D). Infrared thermography revealed that wintering raccoon dogs conserved heat through postural changes such as curling. This behavior resulted in significantly lower average and minimum surface temperatures, even as the localized maximum temperature increased (Figure 1E–H).

Hematological and biochemical analyses supported this systemic shift. Raccoon dogs in winter showed significantly elevated red blood cell (RBC) counts (Figure 1I), suggesting an enhanced oxygen-carrying capacity to support physiological demands during dormancy. Concurrently, total white blood cell (WBC) (Figure 1J) and lymphocyte (LYM) counts were upregulated (Figure 1K), indicating a heightened state of immune vigilance despite metabolic suppression. Notably, beyond these selected parameters, the majority of hematological and biochemical indices did not differ significantly between autumn and winter (Tables S1 and S2), further reflecting a relatively stable internal state during winter sleep. In line with this overall stability, serum triglyceride (TG) (Figure 1L) and glucose levels (Figure 1M) remained unchanged across seasons, highlighting a maintained metabolic homeostasis in raccoon dogs.

Collectively, these findings demonstrate that raccoon dogs achieve a state of metabolic suppression during hibernation by reducing feed intake and core body temperature while maintaining robust systemic homeostasis through the precise regulation of blood glucose and lipids alongside heightened immune vigilance.

### 2.2. Hibernation-Induced Adipocyte Atrophy Coupled with Profound Transcriptional Remodeling in Raccoon Dogs

**Figure 2:**
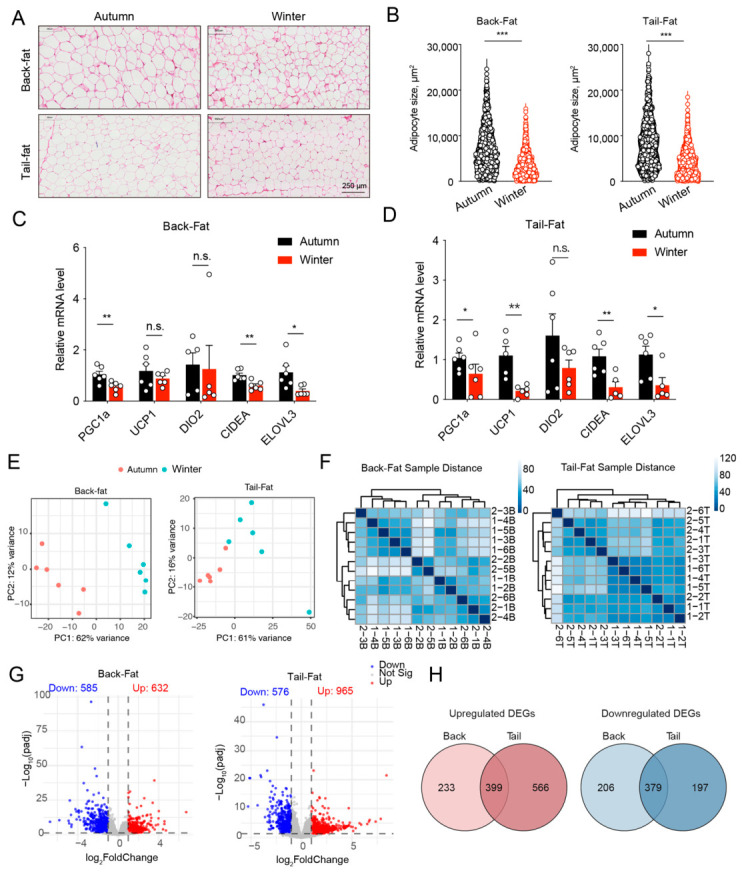
Morphological and transcriptomic landscapes of back-fat and tail-fat depots during hibernation. (**A**), Representative H&E staining of back-fat and tail-fat sections from raccoon dogs in autumn and winter, scale bar: 250 μm. (**B**), Quantitative analysis of adipocyte size in back-fat and tail-fat depots. (**C**,**D**), Relative mRNA expression levels of thermogenic marker genes in back-fat (**C**) and tail-fat (**D**) measured by RT-qPCR, *n* = 5–6. (**E**), PCA of transcriptomic data from back-fat and tail-fat across seasons, *n* = 6. (**F**), Sample distance heatmaps showing the clustering and consistency between autumn and winter groups in back-fat and tail-fat depots. (**G**), Volcano plots illustrating DEGs in back-fat and tail-fat during winter sleep. Blue and red dots represent significantly downregulated and upregulated genes, respectively. (**H**), Venn diagrams showing the overlap of upregulated and downregulated DEGs between back-fat and tail-fat during winter sleep. The left Venn diagram shows commonly and depot-specifically upregulated genes, and the right Venn diagram shows commonly and depot-specifically downregulated genes. Data are presented as mean ± SEM. * *p* < 0.05, ** *p* < 0.01, *** *p* < 0.001. n.s. means not significant.

Back-fat represents a classical subcutaneous WAT depot, whereas tail-fat is a distinct caudal fat depot. In several mammals, tail-associated fat storage functions as an adaptive energy reserve under harsh environmental or nutritional stress [26]. Based on this anatomical and functional distinction, we compared WAT from the back and tail regions of raccoon dogs during autumn and winter to investigate depot-specific adipose adaptations during winter sleep. Hematoxylin and eosin (H&E) staining revealed that adipocytes in both back-fat and tail-fat underwent significant atrophy during hibernation (Figure 2A,B). Notably, no multilocular lipid droplet structures, a characteristic of thermogenic adipocytes, were observed in either depot.

Unlike some deep hibernators that retain thermogenic potential during torpor, the transcriptional profile of raccoon dog adipose tissue indicates the robust inhibition of the browning program. Real-time quantitative PCR analysis demonstrated that the mRNA expression levels of key thermogenic markers, including PGC1a, CIDEA, and ELOVL3, were significantly downregulated in both back-fat (Figure 2C). This suppression was even more pronounced in tail-fat. In this depot, UCP1 was also markedly decreased alongside the aforementioned genes (Figure 2D).

To further explore the molecular mechanisms driving these morphological and metabolic shifts, we performed transcriptome sequencing on both adipose depots. Principal Component Analysis (PCA) and sample distance clustering revealed that winter sleep acts as the primary driver of transcriptomic variance. For both back-fat and tail-fat, PC1 accounted for over 60% of the total variance (62% and 61%, respectively), achieving a complete and distinct segregation between the autumn and winter groups, alongside high intra-group consistency (Figure 2E,F).

Volcano plots illustrated extensive transcriptional remodeling in both WAT depots during winter sleep (Figure 2G). In back-fat, 632 genes were significantly upregulated, and 585 genes were downregulated. In tail-fat, 965 genes were significantly upregulated, and 576 genes were downregulated (Figure 2G). To clarify the directionality of shared transcriptional changes, DEGs were further separated into upregulated and downregulated gene sets. This analysis identified 778 shared DEGs between back-fat and tail-fat, including 399 commonly upregulated genes and 379 commonly downregulated genes during winter sleep (Figure 2H). In contrast, 439 genes were uniquely regulated in back-fat and 763 genes were uniquely regulated in tail-fat (Figure 2H). Together, these results indicate that WAT remodeling during hibernation involves both coordinated seasonal responses and depot-specific transcriptional regulation.

### 2.3. Shared Molecular Signatures and Metabolic Reprogramming of Back-Fat and Tail-Fat Depots

**Figure 3:**
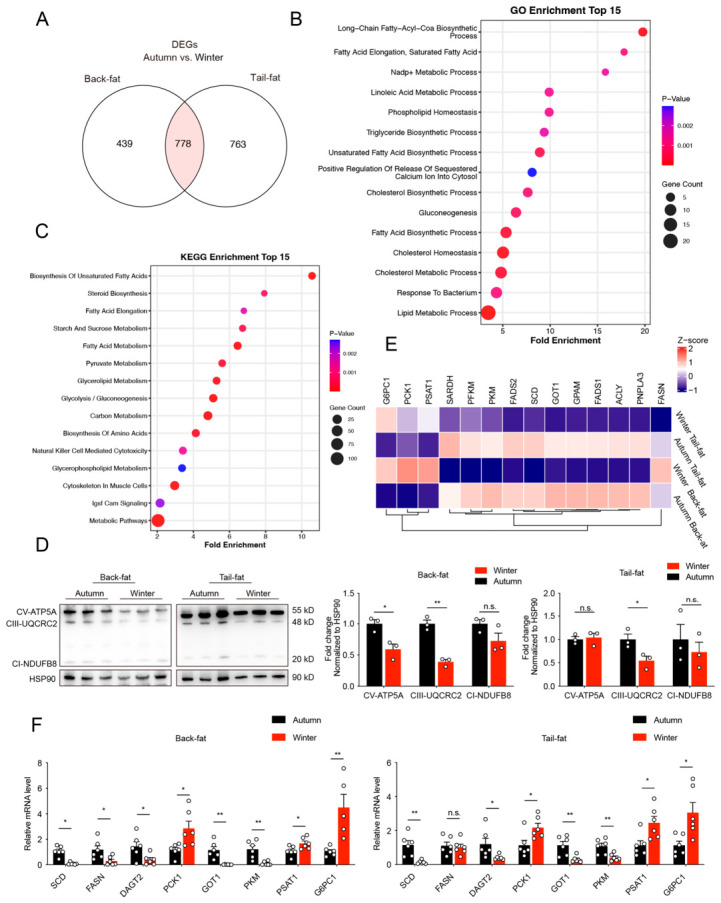
Systemic metabolic reprogramming of core metabolic pathways in WAT during hibernation. (**A**), Venn diagram showing the 778 shared DEGs between back-fat and tail-fat depots during winter sleep. (**B**,**C**), GO (**B**) and KEGG (**C**) enrichment analysis of the 778 shared DEGs. (**D**), Western blot analysis of mitochondrial respiratory chain complex proteins in back-fat and tail-fat depots. HSP90 served as the loading control. (**E**), Heatmap illustrating the transcriptional profiles of key genes involved in fatty acid synthesis, glycolysis, and gluconeogenesis across different depots and seasons. (**F**), RT-qPCR validation of representative genes associated with metabolic suppression and selective regulation in back-fat and tail-fat, *n* = 5–6. Data are presented as mean ± SEM. * *p* < 0.05, ** *p* < 0.01. n.s. means not significant.

To delineate the region-independent molecular signatures of white adipose tissue in response to hibernation, we focused on the 778 shared DEGs between the back-fat and tail-fat depots (Figure 3A). Gene Ontology (GO) and Kyoto Encyclopedia of Genes and Genomes (KEGG) enrichment analyses revealed that these core DEGs were predominantly enriched in crucial metabolic networks, including oxidative phosphorylation, pyruvate metabolism, fatty acid metabolism, and glycolysis (Figure 3B,C).

To validate this metabolic shift at the protein level, we evaluated the expression of mitochondrial respiratory chain complex proteins by Western blotting. Densitometric quantification after normalization to HSP90 showed that several core oxidative phosphorylation-related proteins, including CV-ATP5A, CIII-UQCRC2, and CI-NDUFB8, were reduced in WAT during winter sleep (Figure 3D). These results support the transcriptomic evidence that mitochondrial oxidative metabolism is suppressed in both back-fat and tail-fat during hibernation.

Furthermore, we investigated molecular pathways related to biological macromolecule synthesis. Both heatmap clustering and quantitative validation consistently showed that key genes involved in fatty acid synthesis, amino acid synthesis, and other essential metabolic processes, such as *SCD*, *FASN*, *GOT1*, and *PKM*, were significantly downregulated at the mRNA level during hibernation (Figure 3E,F). Interestingly, amidst this widespread suppression of metabolic and synthetic pathways, *G6PC1*, *PCK1*, and *PSAT1* exhibited a starkly contrasting pattern and were significantly upregulated (Figure 3E,F). Although the functional significance of this pattern requires further investigation, it suggests that WAT metabolism during winter sleep is not uniformly suppressed but may involve selective regulation of specific metabolic nodes.

### 2.4. Characterization of ECM and Vascular Alterations in Back-Fat During Hibernation

**Figure 4:**
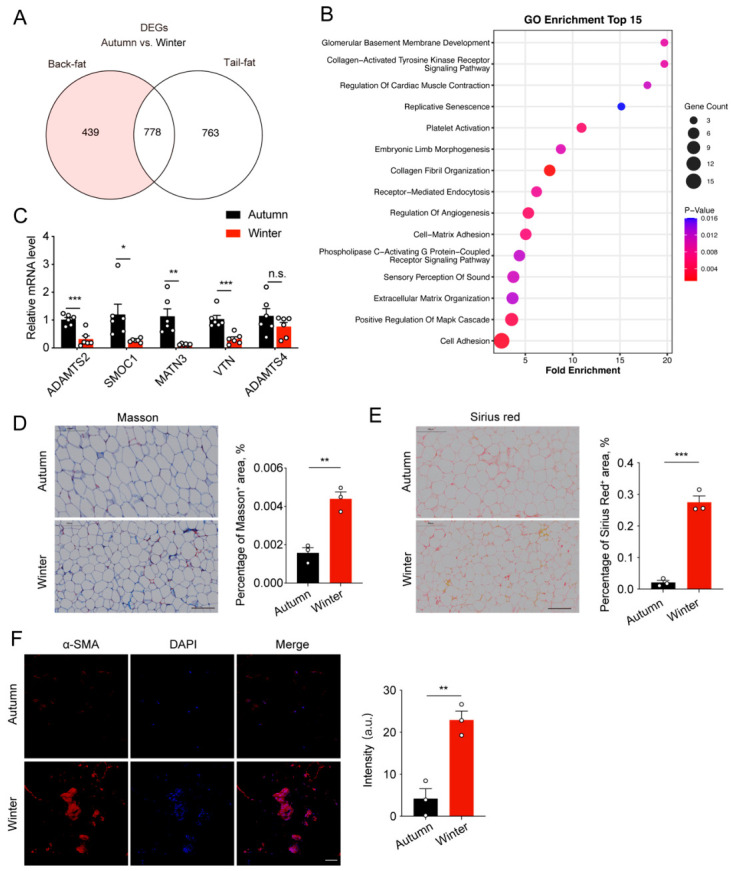
Structural remodeling in back-fat during hibernation. (**A**), Venn diagram highlighting 439 DEGs regulated exclusively in back-fat during winter sleep. (**B**), GO enrichment analysis of the 439 back-fat-specific DEGs. (**C**), RT-qPCR validation of representative genes involved in ECM synthesis and remodeling, *n* = 6. (**D**,**E**), Representative images and quantitative analysis of Masson staining (**D**) and Sirius Red staining (**E**) in back-fat sections, scale bar: 250 μm; *n* = 3. (**F**), Representative immunofluorescence images and quantitative intensity of α-SMA staining (red) in back-fat. DAPI (blue) was used for nuclear staining, scale bar: 100 μm; *n* = 3. Data are presented as mean ± SEM. * *p* < 0.05, ** *p* < 0.01, *** *p* < 0.001. n.s. means not significant.

To elucidate the region-specific adaptations of back-fat during winter sleep, we first performed a comparative transcriptomic analysis. Venn analysis identified 439 DEGs exclusively regulated in the back-fat depot (Figure 4A). GO enrichment analysis of these specific DEGs highlighted a significant overrepresentation of terms related to extracellular matrix (ECM) organization and angiogenesis (Figure 4B).

Specifically, we observed a consistent downregulation of key genes involved in ECM synthesis, assembly, and remodeling, including *ADAMTS2*, *SMOC1*, *MATN3*, *VTN* and *ADAMTS4*. These findings were further validated by RT-qPCR (Figure 4C), indicating a suppressed state of matrix turnover. Interestingly, despite the molecular suppression of remodeling enzymes, histological examination via Masson and Sirius Red staining revealed a seemingly denser collagen network and increased fibrotic appearance in hibernating back-fat compared to the autumn group (Figure 4D,E).

In contrast to the molecular stasis of the ECM, the vascular compartment showed active expansion. GO analysis indicated an enrichment of angiogenic signals (Figure 4B). To confirm this, we performed immunofluorescence staining using α-SMA as a vascular marker. The results demonstrated a significant increase in vascular density within the back-fat of hibernating raccoon dogs (Figure 4F). This expanded microvascular network suggests a compensatory adaptation to maintain basal nutrient delivery under conditions of low blood flow during hibernation.

### 2.5. Immune Cell Profiling and M2 Macrophage Expansion in Tail-Fat During Hibernation

**Figure 5:**
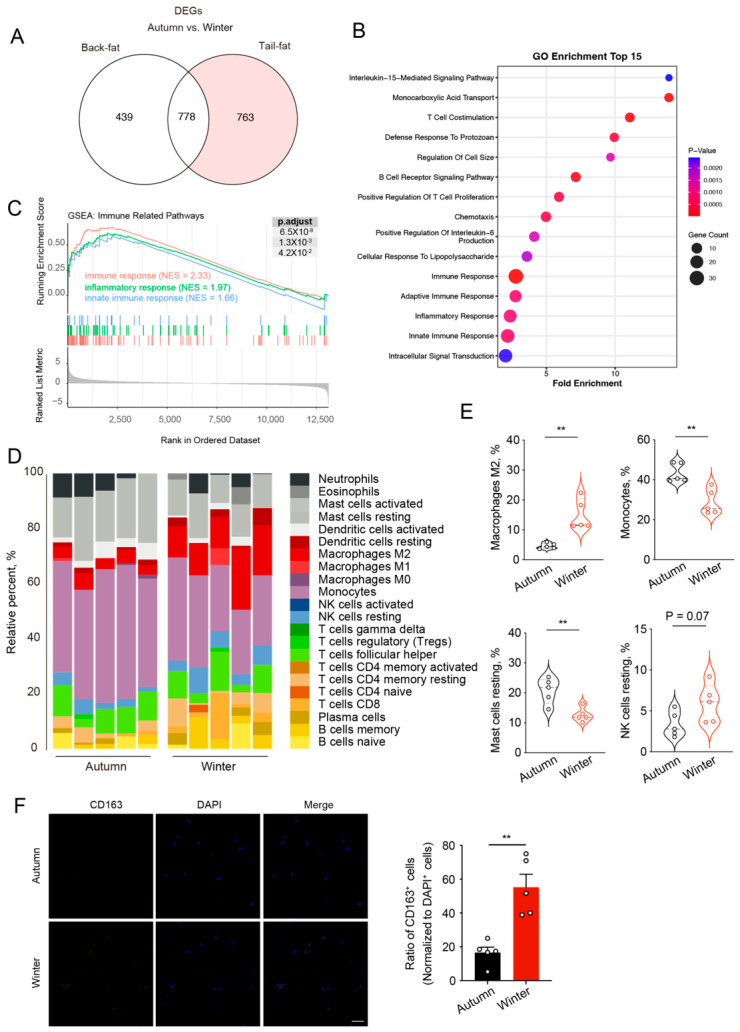
Immune niche remodeling and M2 macrophage infiltration in tail-fat during hibernation. (**A**), Venn diagram highlighting 763 DEGs regulated exclusively in tail-fat during winter sleep. (**B**), GO enrichment analysis of the 763 tail-fat-specific DEGs. (**C**), GSEA plots showing significant enrichment of immune-related pathways in the winter group. (**D**), CIBERSORTx analysis estimating the relative proportions of 22 immune cell types in tail-fat across seasons, *n* = 6. (**E**), Quantitative comparison of specific immune cell populations, *n* = 6. (**F**), Representative immunofluorescence images and quantitative ratio of CD163-positive macrophages (red) in tail-fat sections. DAPI (blue) was used for nuclear staining, scale bar: 100 μm; *n* = 3. Data are presented as mean ± SEM. ** *p* < 0.01.

To explore the regional molecular characteristics of tail-fat, we identified 763 DEGs that were specifically regulated in this depot during winter sleep (Figure 5A). Functional enrichment analysis revealed that these tail-fat-specific DEGs were predominantly associated with inflammatory response, chemotaxis, and innate immune response (Figure 5B). Further analysis using Gene Set Enrichment Analysis (GSEA) confirmed a robust activation of the innate immune system in the winter group, with significant enrichment of pathways involved in pattern recognition and pro-inflammatory signaling (Figure 5C).

To resolve the specific immune cell types contributing to this signature, we performed CIBERSORTx analysis to estimate the infiltration of 22 immune cell types. The results suggested immune niche remodeling in tail-fat during winter sleep (Figure 5D). Specifically, the proportion of M2 macrophages significantly increased, while the abundance of monocytes markedly decreased (Figure 5E). This shift suggests the recruitment of circulating monocytes and their subsequent polarization into an anti-inflammatory M2 phenotype within the adipose tissue. Additionally, we observed a reduction in resting mast cells and resting NK cells in the winter group (Figure 5E), further indicating a transition from basal immune surveillance to a specialized hibernation-related immune state.

Since the LM22 reference matrix was originally developed from human immune cell signatures, the CIBERSORTx analysis was used as an exploratory approach, and the inferred immune cell proportions in raccoon dog adipose tissue should be interpreted with caution. To further evaluate the predicted M2 macrophage-associated remodeling, we performed immunofluorescence staining using CD163, a commonly used marker of M2-like macrophages. In line with this exploratory inference, the tail-fat of hibernating raccoon dogs exhibited a significantly higher density of CD163-positive macrophages compared to the autumn group (Figure 5F). These macrophages were distributed throughout the interstitial spaces between adipocytes. Together with the expression patterns of associated immune markers, these findings suggest that tail-fat undergoes macrophage-associated immune niche remodeling during winter sleep.

## 3. Discussion

Here, we identified raccoon dog shallow hibernation as a natural model of non-thermogenic adipose tissue adaptation. During winter sleep, raccoon dogs experience reduced food intake, body weight loss, and mild hypothermia, yet systemic glucose and lipid homeostasis remain relatively stable. This physiological state differs markedly from pathological metabolic stress, in which adipose tissue remodeling is often accompanied by chronic inflammation, fibrosis, insulin resistance, and metabolic dysfunction. Thus, raccoon dog hibernation provides an opportunity to understand how WAT can undergo extensive seasonal remodeling while preserving tissue and systemic homeostasis.

A central feature of this adaptation is the apparent reliance on WAT-centered, rather than BAT-centered, energy regulation. In classical deep-hibernating rodents, hibernation is closely associated with BAT activation and WAT browning, which support non-shivering thermogenesis [23,27]. By contrast, raccoon dogs show diminished BAT activity during winter sleep [24], and our findings further support a suppressed thermogenic program in WAT. Although adipocyte shrinkage during winter sleep may superficially resemble WAT beiging, classical beiging is typically characterized by multilocular lipid droplets together with the induction of thermogenic genes such as *UCP1*, *PGC1a*, *CIDEA*, and *ELOVL3*. In raccoon dogs, however, smaller adipocytes were not accompanied by clear multilocular lipid droplet formation or thermogenic gene activation, suggesting that this morphology more likely reflects lipid mobilization-associated adipocyte atrophy rather than the emergence of beige adipocytes. This suggests that, in species with limited BAT thermogenic capacity, WAT may assume broader roles beyond lipid storage, including metabolic buffering, structural preservation, and immune regulation. This strategy resembles that of large-bodied hibernators such as bears but is particularly notable in raccoon dogs because their smaller body size would theoretically increase vulnerability to heat loss [16].

The shared transcriptional response of back-fat and tail-fat suggests that metabolic depression is a core WAT program during shallow hibernation. Coordinated suppression of oxidative phosphorylation, glycolysis, fatty acid metabolism, and biosynthetic pathways may reduce unnecessary energy expenditure and limit metabolic imbalance during prolonged food restriction. Rather than representing a passive shutdown, this response appears to be selectively organized, as specific metabolic nodes such as PCK1, G6PC1, and PSAT1 were maintained or activated. These pathways may help preserve carbon flux, prevent the accumulation of metabolic intermediates, and enhance antioxidant capacity during seasonal metabolic transitions [28,29,30,31,32,33,34]. Therefore, raccoon dog WAT appears to combine broad energy conservation with targeted metabolic buffering, which may contribute to the maintenance of systemic homeostasis during winter sleep.

Beyond this shared metabolic depression, different WAT depots also use distinct adaptive strategies. Back-fat appears to adopt a structural remodeling program. In obesity, ECM accumulation and vascular remodeling are often interpreted as signs of pathological fibrosis and tissue dysfunction [2,4,35,36]. However, in the context of hibernation, increased collagen density together with the suppression of several ECM-remodeling genes may reflect matrix compaction or structural stabilization rather than active fibrogenesis. Such remodeling may help preserve adipose tissue architecture during adipocyte shrinkage and lipid mobilization. It may also contribute to local insulation or mechanical protection during winter sleep, providing a structural strategy that complements the suppression of active thermogenesis.

In contrast, tail-fat appears to adopt an immune-protective remodeling program. The enrichment of innate immune pathways and M2 macrophage-associated signatures suggests that tail-fat maintains an active immune niche during winter sleep [37]. Importantly, this immune remodeling occurs in the absence of WAT browning, indicating that macrophage accumulation in this context is unlikely to primarily drive thermogenesis. Instead, M2-like macrophages may support lipid handling, extracellular matrix turnover, anti-inflammatory protection, and tissue repair during prolonged lipid mobilization. This interpretation is consistent with the metabolic preference of alternatively activated macrophages for fatty acid oxidation and oxidative metabolism [38,39,40]. Thus, tail-fat may use local lipid-derived substrates to sustain an immune environment that favors tissue protection rather than inflammatory damage.

These depot-specific responses highlight the functional heterogeneity of WAT during natural metabolic adaptation. Back-fat and tail-fat are not simply interchangeable lipid reservoirs; rather, they appear to perform specialized roles during winter sleep. Back-fat may contribute more to structural preservation, whereas tail-fat may contribute more to immune and tissue-protective regulation. This division of labor provides a framework for understanding how different adipose depots coordinate whole-body energy homeostasis under prolonged metabolic stress.

From a broader biological perspective, raccoon dog hibernation may help distinguish adaptive adipose remodeling from maladaptive adipose dysfunction. In obesity and metabolic disease, adipose tissue remodeling often progresses toward chronic inflammation, fibrosis, hypoxia, and impaired metabolic flexibility. In raccoon dogs, however, seasonal WAT remodeling occurs together with preserved circulating glucose and lipid levels, suggesting that extensive adipose remodeling can be compatible with metabolic health when it is temporally regulated and depot-specific. Understanding the molecular logic of this adaptive remodeling may provide insight into adipose tissue resilience, immunometabolic balance, and metabolic disease resistance.

In summary, raccoon dogs provide a natural model of non-thermogenic WAT adaptation, in which shared metabolic depression and depot-specific remodeling together support metabolic homeostasis during shallow hibernation.

## 4. Materials and Methods

### 4.1. Animals

To minimize potential confounding effects associated with seasonal reproductive cycles and sex hormone fluctuations, only male raccoon dogs (*Nyctereutes procyonoides*) were used in this study. Animals were provided ad libitum access to food and water throughout the experimental period. In October (autumn fat-accumulation phase), raccoon dogs were anesthetized, and small tissue samples of back subcutaneous white adipose tissue (back-fat) and tail white adipose tissue (tail-fat) were surgically collected. The incision sites were subsequently sutured, and animals were allowed to recover. The same sampling procedure was repeated in January (winter hibernation phase) on the same individuals. The order of tissue collection was randomized across animals. Back-fat and tail-fat samples were collected within a short time window during the same surgical procedure to minimize the potential variation caused by sampling time or handling. The animal study protocol was approved by the Ethics Committee of Jilin Agriculture University (protocol code 20250319001 and date of approval 19 March 2025).

### 4.2. Temperature Monitoring

Rectal temperatures of raccoon dogs were measured using a rectal probe connected to a digital thermometer (Jinming Instrument Co., Ltd., Tianjin, China). Measurements were conducted twice in October (autumn fat-accumulation phase) and twice in January (winter hibernation phase). To minimize diurnal variation and environmental confounding factors, all measurements were performed in the early morning at consistent time points.

### 4.3. Infrared Thermographic Imaging

Whole-body infrared thermographic images of raccoon dogs were obtained using an FLIR E54 infrared camera (FLIR Systems, Wilsonville, OR, USA). Imaging was conducted twice in October (autumn fat-accumulation phase) and twice in January (winter hibernation phase), with all measurements performed in the early morning at consistent time points to minimize diurnal variation and environmental confounding factors. Animals were positioned in a standardized posture during imaging to ensure reproducible thermal mapping. Regional average, minimum, and maximum surface temperatures were quantified using FLIR Tools analysis software (https://ignite.flir.com; FLIR Systems, Wilsonville, OR, USA). It should be noted that infrared thermography reflects fur-covered body surface temperature rather than core body temperature. Therefore, the low apparent temperature values detected by infrared imaging likely result from the combined effects of dense fur insulation, low ambient temperature, and imaging geometry.

### 4.4. Hematological and Biochemical Analyses

Blood samples were collected from fasted raccoon dogs in the early morning. For hematological analysis, whole blood was collected into tubes containing ethylenediaminetetraacetic acid (EDTA) as an anticoagulant and immediately analyzed using an automated hematology analyzer. For biochemical analysis, blood was collected into tubes without anticoagulant, allowed to clot at room temperature for 30 min, and then centrifuged at 3000× *g* for 10 min to obtain serum. A full panel of serum biochemical parameters was determined using an automatic biochemical analyzer with available diagnostic kits (IDEXX Laboratories, Inc., Westbrook, ME, USA).

### 4.5. RNA Preparation and Quantitative Real-Time PCR

Total RNA was extracted from adipose tissue samples using TRIzol reagent (Invitrogen, Carlsbad, CA, USA) and further purified with RNeasy Mini spin columns (Qiagen, Hilden, Germany; cat. no. 74106) according to the manufacturer’s instructions. RNA quality and purity were assessed using a NanoDrop 2000 spectrophotometer (Thermo Fisher Scientific, Waltham, MA, USA) by measuring absorbance at 260 and 280 nm. All samples exhibited absorbance ratios (A260/A280) ranging from 1.80 to 2.00. cDNA was synthesized using the PrimeScript RT Reagent Kit with gDNA Eraser (Takara, Tokyo, Japan) following the manufacturer’s protocol. Real-time quantitative PCR was conducted using SYBR Premix Ex Taq (Tli RNaseH Plus; Takara, Tokyo, Japan) on an ABI 7500 detection system (Applied Biosystems, Thermo Fisher Scientific, Foster City, CA, USA) with a reaction volume of 20 μL. All primer sequences are listed in Table S3. Relative gene expression levels of all PCR samples were calculated using the 2^−ΔΔCt^ method. Statistical analysis was performed in GraphPad Prism v6.0.

### 4.6. Histological Analysis

Adipose tissue samples were fixed in 4% paraformaldehyde (PFA) for 48 h, subsequently dehydrated, and embedded in paraffin. Sections (5 μm) were cut and subjected to hematoxylin and eosin (H&E) staining, Masson’s trichrome staining, and Sirius Red staining according to standard protocols. For immunofluorescence staining, frozen sections were prepared and blocked with 5% bovine serum albumin (BSA) for 30 min at room temperature. Sections were then incubated overnight at 4 °C with primary antibodies against α-smooth muscle actin (α-SMA; Abcam, Cambridge, UK; ab7817; 1:200) and CD163 (Novus Biologicals, Centennial, CO, USA; NB110-40686; 1:100). After washing, sections were incubated with corresponding secondary antibodies for 1 h at room temperature. All sections were mounted using Fluoroshield mounting medium with DAPI (Beyotime, Shanghai, China). Imaging was then performed on a laser scanning confocal microscope (LSM 880, Zeiss, Oberkochen, Germany) and an Airscan super-resolution detector.

### 4.7. Western Blot Analysis

Adipose tissue specimens were immediately frozen in liquid nitrogen following collection and maintained at −80 °C until protein extraction. Frozen tissues were mechanically disrupted and solubilized in ice-cold radioimmunoprecipitation assay (RIPA) buffer containing phenylmethylsulfonyl fluoride (PMSF) and a cocktail of protease inhibitors. Following centrifugation at 12,000× *g* for 15 min at 4 °C, the supernatants were collected, and protein yields were quantified via bicinchoninic acid (BCA) assay. For immunoblotting, equal amounts of protein (30 μg per lane) were resolved on 10–12% polyacrylamide gels under denaturing conditions and electrotransferred onto polyvinylidene difluoride (PVDF) membranes. The membranes were then blocked with 5% non-fat dried milk dissolved in TBST for 2 h at room temperature to minimize non-specific binding. Subsequently, the blots were probed overnight at 4 °C with a primary antibody recognizing the OXPHOS (OXPHOS; Abcam, Cambridge, UK; ab110413; 1:2000 dilution) and HSP90 (HSP90; Proteintech, Wuhan, China; 13171-1-AP; 1:1000 dilution) as the loading control. Following primary antibody incubation, membranes were washed extensively and exposed to appropriate HRP-conjugated secondary antibodies for 1 h at ambient temperature. Specific immunoreactive bands were detected using an enhanced chemiluminescence substrate, and band intensities were captured and analyzed densitometrically.

### 4.8. RNA-Sequencing (RNA-seq) and Bioinformatic Analysis

Library construction and RNA-seq were performed by Glbizzia Sequence (Jiaxing, China) using the DNBSEQ-T7 sequencing platform (MGI Tech, Shenzhen, China). Paired-end reads (150 bp) were generated and mapped to the raccoon dog reference genome.

Quality assessment of raw sequencing data was performed using FastQC (v0.11.3), and low-quality reads and sequencing adapters were trimmed using Trimmomatic (v0.39). Clean reads were aligned to the reference genome (*Nyctereutes procyonoides*) using HISAT2 (v2.0.1), and gene expression levels were quantified as fragments per kilobase of transcript per million mapped reads (FPKM) with StringTie (v3.0.1). Differential expression genes between hibernation and control groups were identified using DESeq2 (v1.48.2), with thresholds set at *p*adj. < 0.05 and |Log2(FC)| > 1. PCA and sample distance clustering were performed to evaluate inter-group variation and intra-group consistency. GO and KEGG enrichment analysis of DEGs were performed using the Database for Annotation, Visualization and Integrated Discovery (DAVID) v6.8. GO terms and KEGG pathways were considered significantly enriched at *p*adj. < 0.05. Additionally, the clusterProfiler package (v4.16.0) was utilized to preform GSEA, to identify coordinated changes in predefined gene sets associated with metabolic and immune processes.

### 4.9. Statistical Analysis

All data are presented as mean ± standard error of the mean (SEM) based on a minimum of three biological replicates per group. The normality of data distribution was assessed using the Shapiro–Wilk test. For comparisons between two groups, statistical significance was determined using unpaired two-tailed Student’s *t*-tests. Differences were considered statistically significant at *p* < 0.05. All statistical analyses were performed using GraphPad Prism software (version 9.0, GraphPad Software, San Diego, CA, USA) or R software (version 4.2.0).

## 5. Conclusions

In conclusion, raccoon dogs maintain metabolic homeostasis during shallow hibernation through a non-thermogenic, WAT-centered adaptive strategy. Both back-fat and tail-fat exhibit adipocyte atrophy and shared suppression of core metabolic pathways, while also adopting depot-specific remodeling programs. Back-fat is characterized by structural remodeling involving collagen densification and vascular-associated adaptation, whereas tail-fat undergoes macrophage-associated immune niche remodeling. These findings reveal that WAT depots play coordinated but functionally distinct roles in supporting metabolic stability during winter sleep.

## Data Availability

The raccoon dog adipose RNA-seq data have been deposited in the NGDC GSA database (https://ngdc.cncb.ac.cn/gsa/) under accession number CRA042286. This paper does not report original code. Any additional information required to reanalyze the data reported in this paper is available from the lead contact upon request.

## Supplementary Materials

The following supporting information can be downloaded at https://www.mdpi.com/article/10.3390/ijms27125611/s1.

## Abbreviations

The following abbreviations are used in this manuscript:

WAT	White adipose tissue
BAT	Brown adipose tissue
UCP1	uncoupling protein 1
DEGs	differentially expressed genes
RBC	red blood cell
WBC	white blood cell
lymphocyte	LYM#
TG	triglyceride
PCA	Principal Component Analysis
GO	Gene Ontology
KEGG	Kyoto Encyclopedia of Genes and Genomes
ECM	extracellular matrix
GSEA	Gene Set Enrichment Analysis
