# Supplementary material for: Depot-Specific White Adipose Tissue Remodeling Supports Non-Thermogenic Metabolic Homeostasis During Shallow Hibernation in Raccoon Dogs
Source: Int J Mol Sci. 2026 Jun 22;27(12):5611. doi: 10.3390/ijms27125611 (PMC13299191; doi:10.3390/ijms27125611)
Supplement: Supplementary file 1 [file ijms-27-05611-s001.zip › ijms-4382099-supplementary.pdf]

Supplementary information

Table S1. Hematological analysis in two groups of raccoon dogs.

Trait	Autumn		Winter		
	n	Mean ± SEM	n	Mean ± SEM	*P* value
WBC, 10^9/L	6	17.12 ± 0.98	6	24.35 ± 2.25	0.015 *
LYM, %	6	27.92 ± 3.37	6	45.92 ± 2.36	0.001 **
MID, %	6	9.67 ± 1.35	6	7.63 ± 0.0.39	0.178
GRAN, %	6	62.72 ± 3.45	6	46.45 ± 2.06	0.002 *
LYM, 10^9/L	6	4.82 ± 0.74	6	11.33 ± 1.44	0.002 *
MID, 10^9/L	6	1.67 ± 0.30	6	1.85 ± 0.15	0.598
GRAN, 10^9/L	6	10.63 ± 0.63	6	11.17 ± 0.87	0.631
RBC, 10^12/L	6	6.19 ± 0.10	6	6.80 ± 0.20	0.020 *
HGB, g/L	6	123.33 ± 2.55	6	137.83 ± 4.85	0.025 *
HCT, %	6	38.73 ± 0.69	6	41.48 ± 1.35	0.099
MCV, fL	6	62.70 ± 0.34	6	61.10 ± 0.38	0.011*
MCH, pg	6	19.87 ± 0.21	6	20.23 ± 0.14	0.179
MCHC, g/L	6	317.83 ± 2.01	6	331.50 ± 1.26	0.000 ***
RDW-SD, fL	6	32.80 ± 0.38	6	31.58 ± 0.48	0.074
RDW-CV, %	6	15.87 ± 0.12	6	15.65 ± 0.21	0.388
PLT, 10^9/L	6	420.83 ± 40.58	6	325.83 ± 78.21	0.306
MPV, fL	6	13.18 ± 0.47	6	12.42 ± 1.10	0.538
PDW, %	6	14.53 ± 0.66	6	12.23 ± 1.20	0.123
PCT, %	6	0.56 ± 0.06	6	0.44 ± 0.13	0.455
P-LCR, %	6	18.22 ± 1.08	6	28.93 ± 5.38	0.079 *

Table S2. Hematological analysis in two groups of raccoon dogs.

	Autumn		Winter		
Trait	n	Mean ± SEM	n	Mean ± SEM	*P* value
TP, g/L	6	64.60 ± 2.48	6	72.64 ± 1.81	0.018 *
ALB, g/L	6	41.85 ± 0.46	6	43.32 ± 1.50	0.210
GLO/GLB, g/L	6	22.75 ± 2.10	6	29.22 ± 2.44	0.067
A/G	6	1.95 ± 0.25	6	1.54 ± 0.14	0.180
TBIL, μmol/L	6	3.67 ± 0.43	6	4.74 ± 0.85	0.272
ALT, U/L	6	52.83 ± 25.47	6	39.60 ± 6.05	0.583
AST, U/L	6	55.83 ± 13.74	6	60.40 ± 8.59	0.992
AST/ALT	6	1.41 ± 0.16	6	1.60 ± 0.17	0.670
GGT/γ-GT, U/L	6	0.80 ± 0.19	6	5.30 ± 1.55	0.013 *
ALP/AKP, U/L	6	39.33 ± 2.58	6	26.40 ± 0.81	0.001 **
TBA, μmol/L	6	8.49 ± 0.49	6	9.56 ± 0.78	0.397
CK, U/L	6	238.83 ± 19.52	6	293.20 ± 70.36	0.639
AMY/AMS, U/L	6	1776.83 ± 232.24	6	1793.60 ± 60.42	0.997
TG, mmol/L	6	0.65 ± 0.09	6	0.51 ± 0.06	0.342
TC/CHOL, mmol/L	6	3.00 ± 0.13	6	2.58 ± 0,16	0.110
GLU, mmol/L	6	6.13 ± 0.52	6	6.87 ± 0.45	0.222
Cr/CRE, umol/L	6	58.83 ± 6.47	6	67.60 ± .3.74	0.240
BUN, mmol/L	6	4.95 ± 0.31	6	3.63 ± 0.20	0.009 **
TCO_2, mmol/L	6	24.60 ± 2.19	6	28.40 ± 0.65	0.097
Ca, mmol/L	6	2.74 ± 0.08	6	2.75 ± 0.06	0.806
P/IP, mmol/L	6	1.90 ± 0.14	6	1.44 ± 0.10	0.029 *
Ca×P, mg/dL	6	64.00 ± 3.25	6	49.20 ± 4.34	0.027 *
Mg, mmol/L	6	1.05 ± 0.02	6	1.04 ± 0.05	0.827

Table S3. Gene primers used for qPCR.

Gene	Forward primer (5'-3')	Reverse primer (5'-3')
PGC1a	GGACTCAAGTGGTGCAGTGA	AGTGCATCGAATGAGGGCAA
UCP1	GCCAGGGAGCTAGTTTAGGAA	TAGGCATTGTAAGTGCCGGT
DIO2	CCTCCTCGATGCCTACAAGC	GCTGAGCCAAAGTTGACCAC
CIDEA	TACTGGAGTCAAAGCCCTGC	GAGCATGTACGAGCTGGTGT
ELOVL3	GGCCTTCATCATCCTGCGTA	ACGTGGGGGACTTCACTTTG
B2M	ACTGCTACGTGTCAGGGTTC	GACAGGTCTGTCTGTTCTGCT
SCD	GGAGTCACCGCACCTACAAA	TTTGCGTACAAGCAGCCAAC
FASN	TCCTTCTACCACAAGCTGCG	ATCACATGCACGGACACCTT
DGAT2	AGTACATTGGCTTTGCCCCA	AGGTCTATGTCCTGCTGGGT
PCK1	CACATCTGTGATGGCTCCGA	GCCAACCAGCAGTTGTTGAG
GOT1	TACAATGAGCGTGTGGGCAA	GCTTCTAAGCGTTCCCATTCC
PKM	GGGGCCATAATCGTGCTCA	GGGTCCTTACACACCACAGG
PSAT1	CTGTCCAAGCCAGTGGATGT	GGTCGTCACGGATGATCACA
G6PC1	TCCCGAATCTACCTTGCTGC	CTCTGGATGTGGCGGAAAGT
ADAMTS2	GTACTTCGAGCACGGCGATA	GTGCACCATGCGCTTCATAG
ADAMTS4	GAGGAGCAGTGTGCTGCTTA	AGGGAGTCCCATCTACCACC
VTN	TGCCCAGGTACCTATTCCGA	GAGGTCAGTGAAGGCGTCAA
SMOC1	AAGCCAAGCCCAAGAAGTGT	CCTGTTTGTCCTGGGGGAAG
MATN3	CCAGTGCGCTTTTGGTACAC	GCCCTCGTAGCATTCACAGT
